# Radiation Processing and Characterization of Some Ethylene-propylene-diene Terpolymer/Butyl (Halobutyl) Rubber/Nanosilica Composites

**DOI:** 10.3390/polym12102431

**Published:** 2020-10-21

**Authors:** Elena Manaila, Anton Airinei, Maria Daniela Stelescu, Maria Sonmez, Laurentia Alexandrescu, Gabriela Craciun, Daniela Pamfil, Nicusor Fifere, Cristian-Dragos Varganici, Florica Doroftei, Adrian Bele

**Affiliations:** 1 National Institute for Lasers, Plasma and Radiation Research, 409 Atomistilor Street, 077125 Magurele, Romania; elena.manaila@inflpr.ro (E.M.); gabriela.craciun@inflr.ro (G.C.); 2 Petru Poni Institute of Macromolecular Chemistry, 41A Grigore Ghica Voda Alley, 700487 Iasi, Romania; pamfil.daniela@icmpp.ro (D.P.); fifere.nicusor@icmpp.ro (N.F.); varganici.cristian@icmpp.ro (C.-D.V.); florica.doroftei@icmpp.ro (F.D.); bele.adrian@icmpp.ro (A.B.); 3 National Research and Development Institute for Textile and Leather, Leather and Footwear Institute, 93 Ion Minulescu Street, 031215 Bucharest, Romania; maria.stelescu@icpi.ro (M.D.S.); maria.sonmez@icpi.ro (M.S.); laurentia.alexandrescu@icpi.ro (L.A.)

**Keywords:** EPDM, electron beam irradiation, nanosilica, crosslinking, mechanical properties, contact angle, water uptake

## Abstract

Composites based on ethylene–propylene–diene terpolymer (EPDM)**,** butyl/halobutyl rubber and nanosilica were prepared by melt mixing and subjected to different doses of electron beam irradiation. The effect of irradiation dose on the mechanical properties, morphology, glass transition temperature, thermal stability and water uptake was investigated. The efficiency of the crosslinking by electron beam irradiation was analyzed by Charlesby–Pinner parameter evaluation and crosslink density measurements. The scanning electron microscopy data showed a good dispersion of nanosilica in the rubber matrix. An improvement in hardness and 100% modulus was revealed by increasing irradiation dose up to 150 kGy. The interaction between polymer matrix and nanosilica was analyzed using the Kraus equation. Additionally, these results indicated that the mechanical properties, surface characteristics, and water uptake were dependent on crosslink characteristics.

## 1. Introduction

Ethylene–propylene–diene terpolymer (EPDM) is a widely used rubber with superior resistance against heat, ozone, chemical ageing, weathering. Additionally, EPDM exhibits excellent electrical properties, good mechanical properties, resistance to various aggressive chemicals (acids, alkalies, phosphate esters or ketones), good adhesion characteristics and good low-temperature properties due to its saturated and stable polymer backbone structure [1,2,3,4]. EPDM has found increasing applications in automotive sealing systems, roofing membranes, wire and cable insulation coverings, building profiles, insulation layers, belts, etc. [5,6,7]. At the same time, some nanofillers can be included in the EPDM rubber matrix to improve the mechanical and processing characteristics as compared to the conventional fillers [8,9]. Among the rigid nanofillers, nanosilica is commonly utilized as a reinforcing agent in elastomeric and thermoplastic compositions being a heat-resistant, electrical and thermal insulator. The incorporation of small amounts of nanosilica in polymers leads to the improvement of mechanical properties, higher resistance to abrasion, corrosion or scratching, superior fatigue resistance and good adhesion properties [10,11,12,13,14]. As a matter of fact, the concentration and dispersion of nanofiller play an important role in the enhancement of the performance of the polymer composites. Additionally, the performances of polymer composites are determined by the interaction at the interface between nanofiller and polymer matrix [15].

The resistance of EPDM composites to higher temperatures is determined to a great extent on the applied vulcanization method. The EPDM compounds vulcanized with a sulphur cure system by the formation of polysulfide crosslinks, (–C–S_x_–C–), can be utilized for continuous service up to 130 °C. The monosulfide crosslinks, (–C–S–C–), created by sulphur donors such as thiuram disulfide present better stability at higher temperatures than the polysulfide bonds. Using the vulcanization by peroxides or ionizing radiations more resistant –C–C– bonds can be created. In this case, the vulcanized EPDM composition can be used up to 150 °C [16,17]. In order to withstand at a temperature of 150 °C, heat resistant blends based on EPDM and butyl rubber (IIR) can be employed [18,19]. However, in this case, the compounds with butyl rubber or halogenated butyl rubber with high impermeability to gases can produce some processing problems due to the formation of blisters during the vulcanization processes of the finished product [20,21]. These aspects can be exceeded by crosslinking of the composites using electron beam irradiation. This irradiation process takes place at room temperature. The vulcanization by accelerated electron beams has proved many positive results by comparison with the conventional curing systems occurring at high temperatures, using sulphur and vulcanization accelerators when many toxic gaseous products are evolved. Electron beam crosslinking has a series of advantages over the traditional curing procedures namely no polymer degradation due to high temperature, since the electron beam crosslinking occurs at room temperature, with high crosslinking degree, extremely short curing cycles, being an ecological procedure leading to better physical–mechanical properties of the materials [22,23]. It was previously reported that in the crosslinking process with peroxides or ionizing radiations the adding of small amounts of some polyfunctional monomers such as trimethylolpropane triacrylate leads to an increase in the crosslinking degree, to the decrease in crosslinking time and irradiation dose [24,25]. In this way, the irradiation crosslinking can be also utilized for polymer composites in which one component tends to degrade at higher irradiation doses namely butyl rubber or halobutyl rubber [26].

In this study, affordable composites including EPDM/butyl rubber or halobutyl rubber in presence of nanosilica were prepared, irradiated with different accelerated electron beam doses to induce their crosslinking. The mechanical, thermal, morphological and physical properties of the developed composites were analyzed. Additionally, interactions between polymer matrix and nanosilica were discussed.

## 2. Materials and Methods

### 2.1. Materials

EPDM rubber Nordel 4760 (made of 70% ethylene, 4.9% 5-ethylidene norbornene (ENB), Mooney viscosity: 70 ML_1+4_ at 120 °C, crystallinity degree of 10%) was obtained from Dow Chemical Company, Washington, Midland, MI, USA. Butyl rubber (Butyl 268, Mooney viscosity: 521 ML_1+8_ at 125 °C, unsaturation degree of 1.70 mol%), chlorobutyl rubber (Chlorobutyl HT 1066, Mooney viscosity: 38 ML_1+8_ at 125 °C, unsaturation degree of 1.26 mol%) and bromobutyl rubber (Bromobutyl 2222, Mooney viscosity: 32 ML_1+8_ at 125 °C, unsaturation degree of 1.03 mol%) were provided by Exxon Mobil Chemicals, Machelen, Belgium. Nanosilica Aerosil 200 (specific contact surface of 175–225 m^2^/g, pH 3.7–4.5, density of 50 g/cm^3^ and SiO_2_ content > 99.8%) was obtained from Evonik, Degussa, Germany. Trimethylolpropane trimethacrylate Luvomaxx TMPT DL 25 (ash content of 22%, pH 9.2, density of 1.36 g/cm^3^, 75.63% active ingredient) was utilized as polyfunctional monomer. Irganox 1010 (pentaeritritol tetrakis(3-(3,5-di-tert-butyl-4-hydroxy) propionate) (98 % active ingredient) was purchased from BASF Schweiz, Basel, Switzerland.

### 2.2. Preparationof EPDM Composites

The melt mixing of the EPDM butyl/halobutyl rubber and nanosilica was performed in a Brabender Plasticorder type 828703 V230 (Brabender GmbH & Co KG, Duisburg, Germany) at high temperature (160–190 °C) for a mixing time of 10 min and rotor speed of 30–150 rpm. The composition and codes of the resulting EPDM composites are given in Table 1. For ensuring the uniform mixing of components, a laboratory electrical heated roller mill with the working parameters: temperature 60–80 °C, friction 1:1, mixing time 7 min was utilized. Thus, sheets of 2–4 mm thickness were prepared. The test specimens were cut and pressed in a laboratory hydraulic press Fortune Presses, model TP 600, (Fontijine Grotness, Vlaardingen, The Netherlands) at 160 °C, pressing force of 300 kN, preheating time of 1 min and molding time of 5 min.

Irradiation was carried out using an ALID7 electron beam accelerator at the following doses: 20, 50, 100 and 150 kGy, respectively, at room temperature. The values of the working parameters during irradiation were electron beam energy E_EB_ = 5.5 MeV, EB peak current I_EB_ = 26 mA, maximum output power P_EB_ = 134 W, fixed pulse duration τ_EB_ = 3.75 μs and repetition frequency f_EB_ = 50 Hz. Rubber samples in a rectangular shape of 100 mm × 100 mm × 2 mm were exposed to electron beam irradiation.

### 2.3. Measurements

The tensile mechanical tests were performed on a Schopper tensile tester (Veb Thuringer Industriewerk Rauenstein, Leipzig, Germania) using a testing speed of 460 mm/min on dumbbell-shaped specimens according to the conditions described in ISO 37/2020. The hardness, in units of Shore A, was determined using hardness tester according to ISO 48-4/2018. The elasticity was measured with a Schob test apparatus (Veb Thuringer Industriewerk Rauenstein, Leipzig, Germania) on 6 mm thick samples based on ISO 4662/2017.

FTIR absorption spectra were acquired with a Perkin Elmer FT-IR Spectrum 100 (PerkinElmer, Inc, Shelton, CT, USA) coupled to an attenuated total reflection (ATR) attachment (diamond/ZnSe crystal). On each sample, 10 scans were measured in the spectral range of 400–600 cm^−1^ at a resolution of 4 cm^−1^.

Thermogravimetric analyses (TGA) were undertaken on a STA 449F1 Jupiter instrument (Netzsch, Germany) employing 15 mg of sample. The samples were heated in the range 30–700 °C in nitrogen atmosphere at a rate of 50 mL/min, in open Al_2_O_3_ crucibles.

Differential scanning calorimetry (DSC) analysis was carried out in a DSC 200F3 Maya (Netzsch, Germany) under a nitrogen flow rate of 50 mL/min. Heating and cooling rates of 10 °C min^−1^ and −10 °C min^−1^, respectively were applied. An amount of 10 mg of each sample was heated in aluminium crucibles. The device was calibrated with an indium standard.

The gel content of electron beam irradiated composites was determined by extraction with toluene for 24 h. Test samples of 1.0 cm × 1.0 cm were initially weighted (w_1_) and immersed in toluene at room temperature until the attainment of swelling equilibrium. Then, the samples were dried in air for 6 days and finally weighted to obtain the dried mass (w_3_). The gel fraction was calculated according to the relation:

(1) $$\mathrm{Gel}\;\mathrm{fraction}=\frac{w_{3}}{w_{1}}\times 100$$

where w_1_ and w_3_ are the initial weight before extraction and the weight of the dried sample after toluene extraction. The values reported for the gel fraction are the mean value of five measurements.

The crosslinking density was calculated based on the equilibrium swelling in toluene applying the Flory–Rehner equation for tetrafunctional networks [27]. The determination method was presented in our previous studies [19,28].

The dynamic water vapor sorption capacity was determined with a fully automated gravimetric analyzer IGAsorp from Hiden Analytical, Warrington, UK. This analyzer is equipped with an ultrasensitive microbalance measuring changes in the sample mass as a function of the relative humidity (RH) and temperature. The measurements were made at atmospheric pressure by passing a humified stream of gas over the sample. Samples were dried under nitrogen flow (250 mL/min) until the sample weight was stable at RH < 1%. Isothermal determinations were carried out at 25 °C in the relative humidity range of 0–80%, with 10% humidity steps and a pre-established equilibrium time between 10 and 15 min.

The static contact angle measurements of EPDM composites were made by following the sessile drop method at room temperature. A contact angle instrument from KSV Instruments, Helsinki, Finland of CAM 200 type was used for this purpose. A liquid droplet of 1 L was placed on the sample surface and the reported results represent the mean value of 7 replicates at different locations on the sample surface by fitting the drop profile using Young–Laplace equation. For the calculation of the total surface free energy (γ_lv_^TOT^), which is the sum of the polar (γ_sv_^AB^) and dispersive (γ_sv_^LW^) components it was necessary to determine the contact angle in two polar liquids (twice distilled water and formamide) and in one completely dispersive liquid (diiodomethane) with known values of the polar and dispersive components according to Van Oss and Good relation [29,30]

(2) $$\left(1+\cos\theta \right)\cdot \gamma_{1V}^{\mathrm{TOT}}=2\cdot \left(\sqrt{\gamma_{\mathrm{SV}}^{\mathrm{LW}}\cdot \gamma_{1V}^{\mathrm{LW}}}+\sqrt{\gamma_{\mathrm{SV}}^{+}\cdot \gamma_{1V}^{-}}+\sqrt{\gamma_{\mathrm{SV}}^{-}\cdot \gamma_{1V}^{+}}\right)$$

(3) $$\gamma_{\mathrm{SV}}^{\mathrm{AB}}=2\cdot \sqrt{\gamma_{\mathrm{SV}}^{+}\cdot \gamma_{\mathrm{SV}}^{-}}$$

where θ is the static contact angle, γ_lv_^TOT^ is the liquid total surface tension, γ_sv_^AB^ is polar Lewis acid–base interaction, γ_lv_^LW^ and γ_sv_^LW^ are the apolar (dispersive) Lifshitz–van der Waals components of the liquid and solid, respectively, γ_lv_*^−^* and γ_sv_*^−^* represent the electron donor (Lewis base) parts, γ_sv_*^+^* and γ_lv_*^+^* are the electron acceptor (Lewis acid) contributions of the solid (s) and the liquid (l) phases as indicated by the subscripts. The subscripts “lv” and “sv” denote the interfacial liquid–vapor and surface–vapor tensions, respectively.

The morphology of the selected composites before and after the electron beam irradiation at different doses was examined by a Quanta 200 scanning electron microscope (FEI, Brno, The Czech Republic) operating at 20 kV in low vacuum mode with a secondary electron detector LFD.

## 3. Results and Discussion

**Figure 1:**
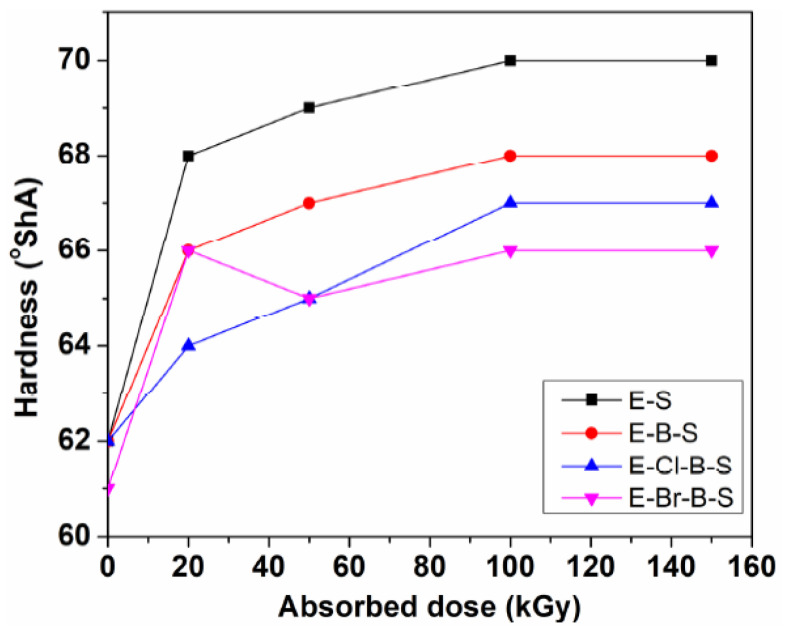
Variation of the hardness of EPDM composites regarding to the irradiation dose.

**Figure 2:**
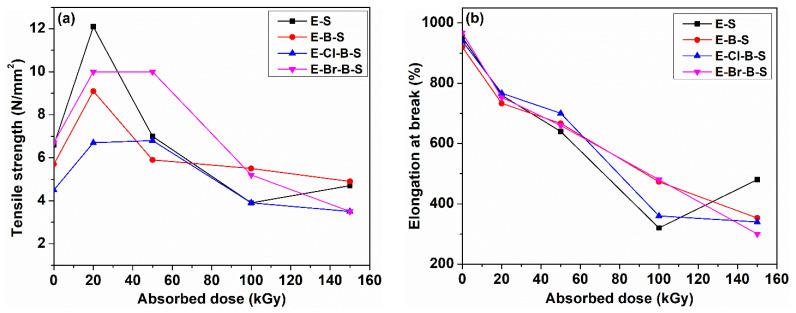

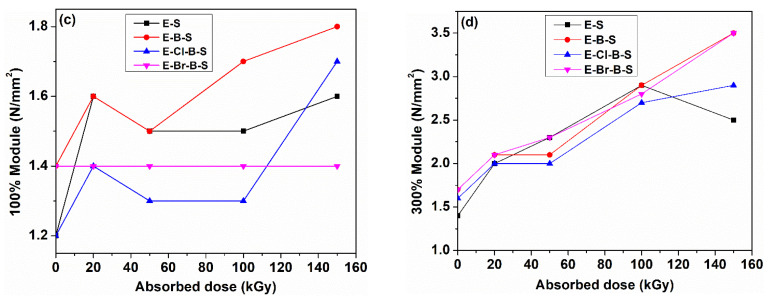
Tensile properties of EPDM composite against the electron irradiation dose: (**a**) tensile strength, (**b**) elongation at break, (**c**) 100% modulus, (**d**) 300% modulus.

**Figure 3:**
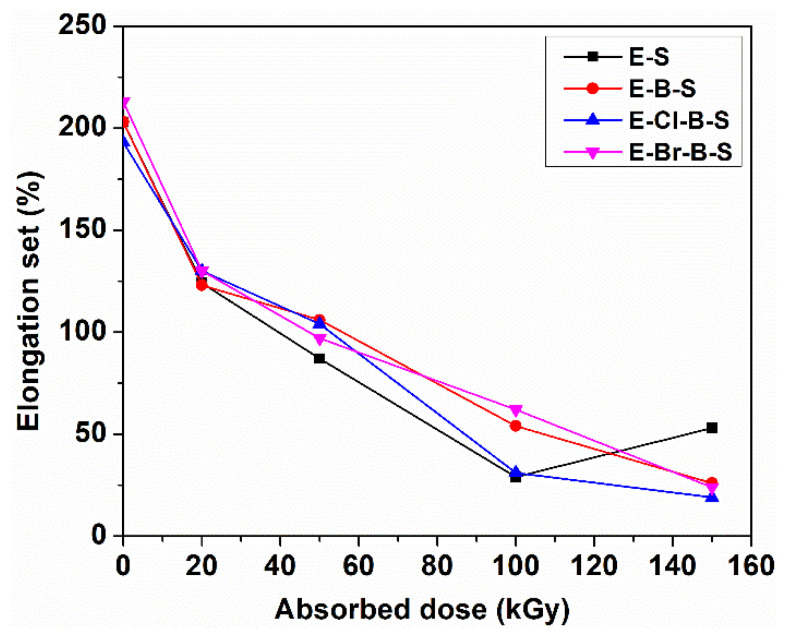
Elongation set at different electron beam irradiation dose.

**Figure 4:**
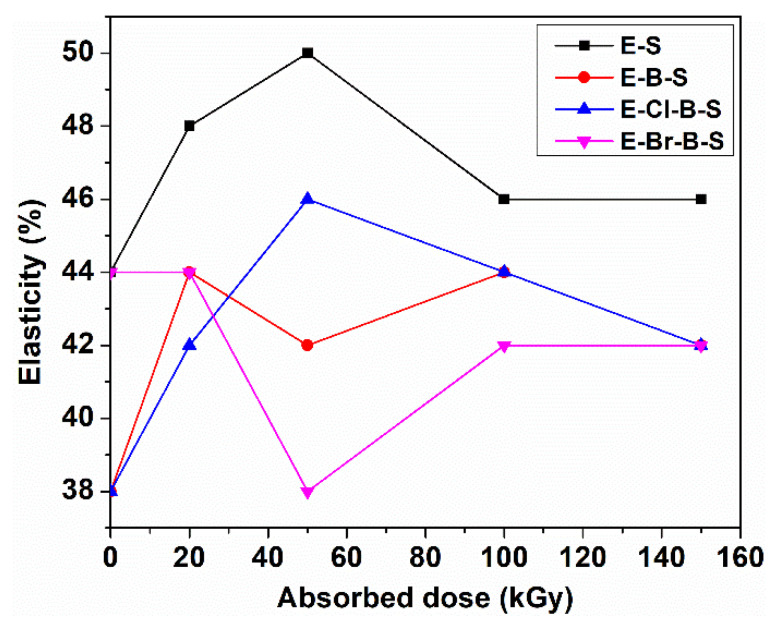
Elasticity as a function of irradiation dose.

The different mechanical characteristics of the EPDM/butyl rubber/nanosilica composites including hardness, tensile strength, elongation at break, 100% modulus, 300% modulus, elongation set and elasticity are presented in Figure 1, Figure 2, Figure 3 and Figure 4 as a function of the irradiation dose. As shown in Figure 1 and Figure 2c,d, it is observed that the hardness, 100% modulus and 300% modulus are improved as the electron beam irradiation dose increases, whereas the elongation at break and elongation set are reduced at higher irradiation doses (Figure 2b and Figure 3). As it can be seen from Figure 1, the highest increase in the hardness was observed for EPDM/nanosilica composite E-S. The hardness varies from 62° ShA for a non-irradiated sample to 70° ShA at an electron beam irradiation dose of 150 kGy. The hardness of EPDM/butyl rubber/nanosilica composites decreases in the following order: E-S > E-B-S > E-Cl-B-S > E-Br-B-S. The improvement of the hardness can be due to a better rubber–silica interaction as well as to the crosslinking process caused by the electron beam irradiation.

The tensile strength of EPDM composites exhibits an increasing trend up to a 20 kGy then decreases at higher doses up to 150 kGy. The highest values of the tensile strength were recorded for E-S and E-Br-B-S samples irradiated with electron beam doses of 20 kGy (Figure 2a). The values of tensile strength of the EPDM/butyl rubber/nanosilica were significantly higher than those of EPDM composites without nanosilica [19]. The incorporation of the nanosilica in the rubber matrix determines an improvement of the tensile characteristics, whereas the presence of butyl rubber or Br butyl halogenated rubber leads to a slight decrease in the tensile strength for irradiation doses of 20 and 50 kGy, due to the strong polar character of the bromobutyl units [19]. The lower values of sample E-Cl-B-S at different irradiation doses can be connected with the ability of chlorine radical to abstract hydrogen from the main polymer chain. The radicals formed from the hydrogen abstraction are known to give rearrangements with the chain scission [25] during electron beam irradiation. The decrease in tensile strength can be caused by a higher crosslink density at high irradiation doses.

When the irradiation dose increased, 100% modulus presented variations between 0% for E-Br-B-S sample and 42% for E-Cl-B-S (Figure 2c), whereas 300% modulus exhibited higher increases: 81% for E-Cl-B-S, 106% for E-B-S and E-Br-B-S and 107% for E-S, respectively. If the irradiation dose increases, the physical bondings from polymer are replaced by more resistant –C–C– bonds due to the crosslinking process. Thus, for an elongation of 100% (100% modulus) small variations of the applied force are necessary, while at an elongation of 300% the applied force increases significantly. For the E-Br-B-S sample due to the improvement of rubber–rubber and rubber–nanosilica compatibility, modifications of the 100% modulus are not practically observed, but an elongation of 300% determines important increases of the 300% modulus values as the irradiation dose increases as a result of the crosslinking by the increase in the number of –C–C– bonds among the polymer chains.

The effect of irradiation dose on the elongation at break and elongation set of EPDM/butyl rubber/nanosilica composites is shown in Figure 2b and Figure 3. It can be seen that both the elongation at break and the elongation set decreased rapidly with the increase in irradiation dose. The decreasing trend of these parameters can be attributed to the crosslinking process under electron beam irradiation leading to the stiffness of EPDM composites which restrict the mobility of the polymer chains [31].

An increase in the elongation set was observed at 150 kGy for sample E-S (Figure 3). The increase in irradiation dose causes an increase in the crosslink density leading to the modification of the sample structure. In this way, the return to the initial state of the sample after a force was applied can be blocked which leads to the increase in elongation set. We must mention that the values of the elongation set at irradiation doses over 100 kGy are superior to those obtained by classical crosslinking [3].

The elasticity increases initially to a 50 kGy dose, and then a decreasing trend was observed at higher irradiation doses (Figure 4), except composite E-Br-B-S, with the lowest values of the elasticity.

**Figure 5:**
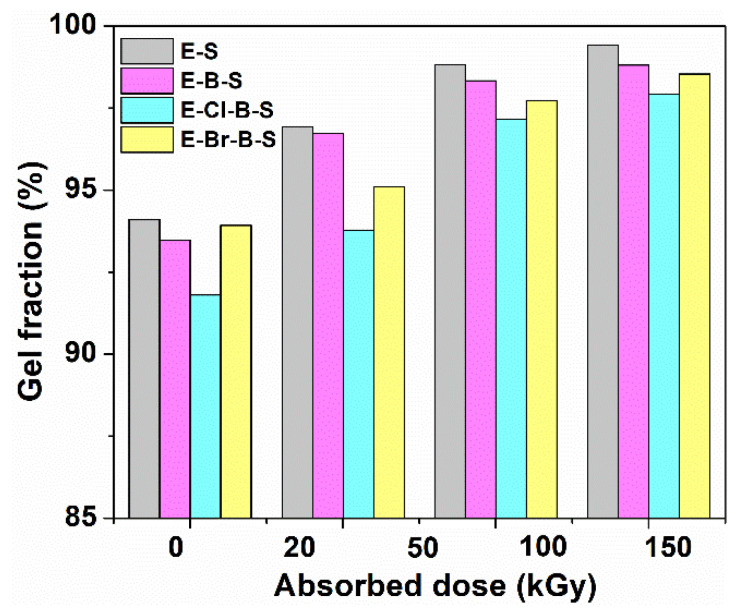
Variation in gel content for EPDM composites with irradiation dose.

**Figure 6:**
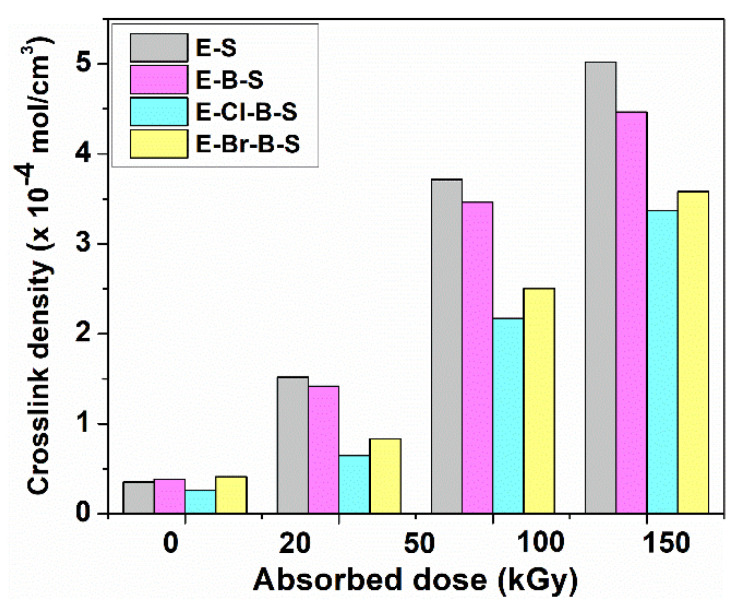
Dependence of the crosslink density on the irradiation dose for the investigated samples.

Figure 5 displays the change in the gel content of EPDM composites in electron beam irradiation. The gel content of EPDM composites increases as the irradiation dose increases. The E-S and E-B-S composites show higher gel content as compared to the composites containing halogenated butyl rubber. High levels of the gel content of 99.4% and 98.8%, respectively were obtained for E-S and E-S-B samples at an irradiation dose of 150 kGy, whereas the lowest gel content was obtained for samples containing chlorobutyl rubber (E-Cl-B-S) in accordance with the lower values of the crosslinking degree for E-C-B-S sample (Figure 6).

To realize insight into the electron beam irradiation-induced crosslinking density determinations were performed using the Flory–Rehner equation. The dependence of crosslink density on the electron beam irradiation dose is illustrated in Figure 6. As can be seen from this figure, at lower irradiation doses there is not much difference among the values of crosslink densities of these composites, but at higher doses, the crosslink densities increase significantly. On the other hand, the gel content values attained practically a plateau at 100 and 150 kGy, respectively, for all samples, the crosslink density kept increasing, the highest values were noticed for E-S and E-B-S samples at 150 kGy. The composites containing halogenated butyl rubber reveal lower crosslink densities due to breaking the C-halogen bonds during irradiation leading to the polymer chain breaking in agreement with previously reported papers [19,25,32].

**Figure 7:**
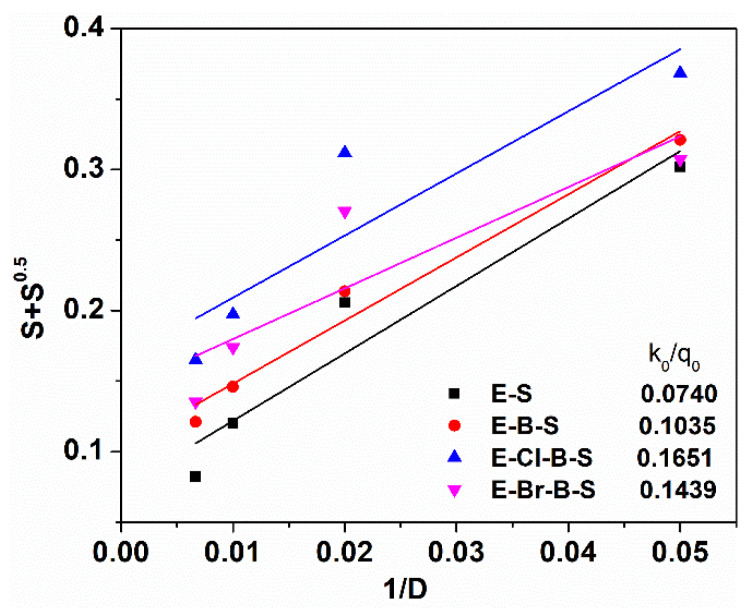
Charlesby–Pinner plots for EPDM composites at different irradiation doses.

To quantitatively estimate whether the crosslinking or the chain scission reaction were more predominant during electron beam irradiation of the EPDM based composites, the Charlesby–Pinner approach was used [6,33]. This expression provides a relationship between sol fraction and irradiation dose:

S + S^0.5^ = (p_0_/q_0_) + (1/αP_n_D) (4)

where D denotes the irradiation dose in kGy, S represents the sol fraction, p_0_ is the average number of chain scissions per monomer unit per dose unit, q_0_ is the crosslinking density per dose unit, P_n_ is the number-average degree of polymerization and = q_0_/2. As shown in Figure 7, the experimental plot of (S + S^0.5^) as a function of irradiation dose is in good agreement with the relation (4). From the intercept (p_0_/q_0_) and the slope (1/q_0_), the values p_0_ and q_0_ were calculated. The values of p_0_/q_0_ ratio resulting from the linear fitting in Figure 7 are smaller than 1 for all EPDM-based composites which suggest that the polymer chains predominantly underwent a crosslinking reaction rather than a scission process. From the data evidenced in Figure 7, it can be seen that the crosslinking extent increased for samples E-S and E-B-S, which were efficiently crosslinked by electron beam irradiation, on the other hand, the ratio p_0_/q_0_ was increased in presence of halogenated butyl rubber in the composite. Despite the dominant crosslinking process in these composites, it was possible that the chain scission reaction to occur and to affect the mechanical properties.

The interaction between polymer matrix and nanosilica was evidenced using the Kraus equation [34,35]:

V_ro_ = 1− m [f/1 − f] (5)

where V_ro_ and V_rf_ represent the volume fraction of rubber in vulcanized state and of rubber in the solvent swollen filled sample, m is the polymer-filler interaction parameter, f represents the volume fraction of filler.

The ratio V_ro_/V_rf_ gives information about the restriction degree of the swelling of the polymer matrix due to the presence of filler [36,37]. The volume fraction of rubber in the swollen sample, V_rf_, was estimated according to the following expression [35]:

V_rf_ = (D − fT)/ ρ_r_ / (D – fT)/ ρ_r_ + A_s_/ρ_s_ (6)

where and ρ_r_ and ρ_s_ denote the densities of EPDM rubber and of solvent, D is the deswollen weight of the sample, T is the initial weight of the rubber sample, A_s_ represents the amount of adsorbed solvent at swelling equilibrium, f is the volume fraction of filler.

The values of V_ro_ and V_rf_ determined according to Equations (5) and (6) are listed in Table 2 for EPDM composites irradiated at different electron beam doses. It can be seen from Table 2 that the equilibrium solvent uptake of EPDM samples decreases as the electron beam irradiation dose increases, leading to an increase in V_rf_ parameter, and hence a corresponding decrease in the ratio V_ro/_V_rf_, since V_ro_ is a constant. This fact can be connected with an enhanced nanofiller–rubber matrix interaction in the composite due to a good fitting with the Kraus model and the ratio V_ro_/V_rf_ < 1 for irradiation doses higher than 20 kGy. Since a better bonding between the rubber matrix and nanosilica, a stronger interface was developed which restricts the entry of the solvent at higher irradiation doses due to the presence of the crosslinks. However, the incorporation of halogenated butyl rubber in the EPDM matrix determines an increase in the V_ro/_V_rf_, due to the appearance of repulsing forces between chlorine or bromine ions and nanosilica with a pH of 3.5–4.0. As the irradiation dose increases the solvent uptake decreases (Table 2).

**Figure 8:**
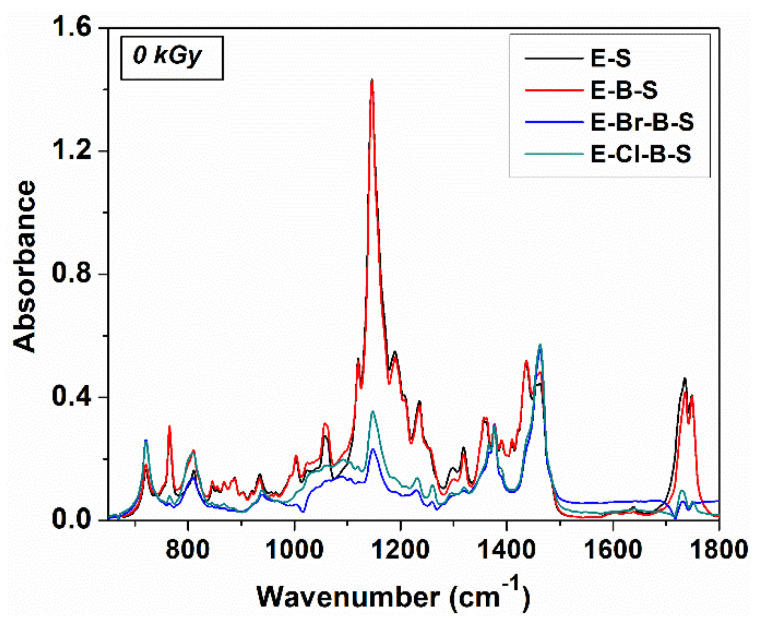
FTIR spectra of non-irradiated EPDM based composites.

**Figure 9:**
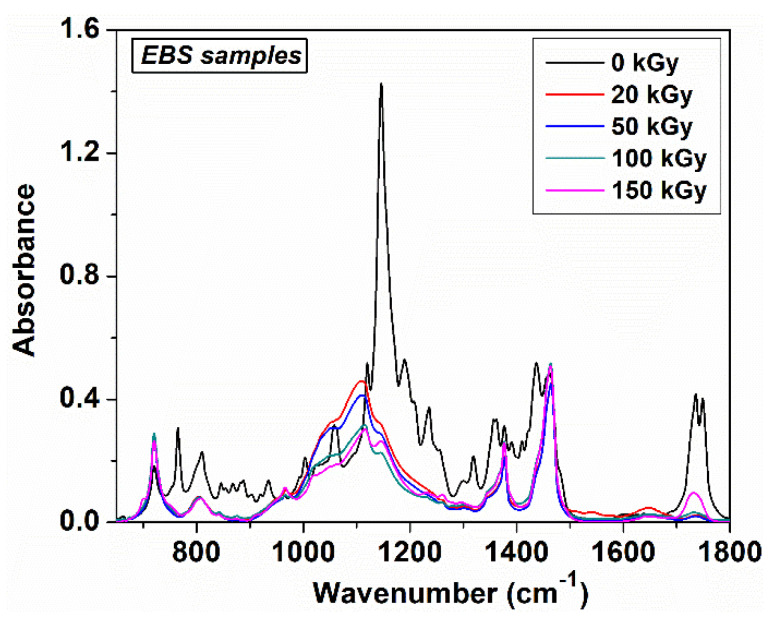
FTIR spectra of electron beam irradiated E-B-S composites at different irradiation doses.

**Figure 10:**
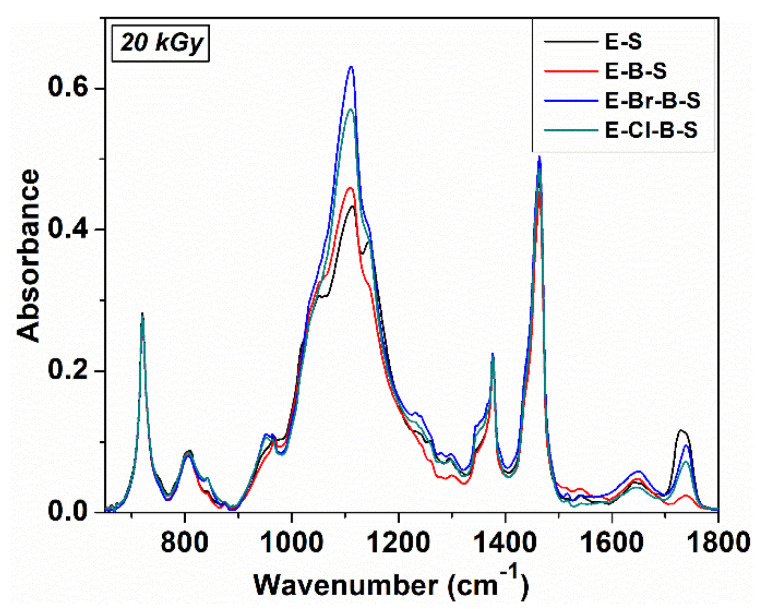
FTIR spectra of EPDM/butyl rubber/silica composites after irradiation at a dose of 20 kGy.

The FTIR absorption spectra of non-irradiated and irradiated EPDM/rubber/nanosilica composites are displayed in Figure 8, Figure 9 and Figure 10. As can be seen, the EPDM can be identified by the absorption bands around 1456 cm^−1^ assigned to –CH_2_ scissoring vibrations, 1377 cm^−1^ due to C–H bending vibration of –CH_3_ groups, 722 cm^−1^ due to –CH_2_ groups rocking vibrations. Additionally, two intense absorption bands at 2920 and 2850 cm^−1^ are ascribed to asymmetric and symmetric C–H stretching vibrations, corresponding to the saturated hydrocarbon backbone [19,38,39,40]. The structure of the EPDM chain can change depending on the relative tactic placement of the adjacent head to tail propylene units and their distribution along the polymer chain, namely isolated or head to tail propylene units, –CH_2_–CH(CH_3_)–CH_2_– at 1159 cm^−1^, head to tail propylene units, –CH_2_–CH(CH_3_)–CH(CH_3_–CH_2_ at 1120 cm^−1^ and side ethylene groups, –CH–(CH_2_–CH_3_) at 767 cm^−1^ which can be used to identify the crystalline ethylene blocks [40,41]. The absorption band around 767 cm^−1^ was influenced by adding nanosilica or butyl/butyl halogenated rubber and it disappeared practically during electron beam irradiation (Figure 9). On the other hand, the absorption bands in the range 1400–1200 cm^−1^ of the butyl/butyl halogenated rubbers are overlapped with the absorption bands resulting from EPDM. The specific absorption bands for TMPT grafted onto EPDM were observed around 1723 and 1638 cm^−1^ which originated from the >C–O stretching vibration and the C=C stretching vibration [42].

By adding nanosilica to EPDM/butyl rubber composites, a very strong absorption band was found around 1146 cm^−1^ due to the Si–O stretching vibration out of plane, while the absorption band at 1057 cm^−1^ can be assigned to Si–O vibration in-plane [43], for E-S and E-B-S samples, whereas in the composites containing halobutyl rubbers, this absorption band decreases in intensity (Figure 9). The difference between the IR absorption spectra of composites without and with halobutyl rubber is confirmed by the values of interaction parameter (Table 2) where an increase in the V_ro_/V_rf_ ratio was observed for halogenated samples.

The electron beam irradiation determines a significant decrease in the intensity of the absorption band at 1735 nm (Figure 10) due to the crosslinking process, in agreement with previous data [44] because the double bonds from TMPT are consumed during the irradiation process. The significant change of the IR absorption bands of the irradiated EPDM composites was observed at lower irradiation doses of 20 kGy (Figure 10), the further increase in the irradiation dose determines small decreases of the intensity in the absorption bands, the absorption profile remains practically unchanged. This fact suggests the consumption of double bonds and the crosslinking at an irradiation dose of 20 kGy, as well as the stability of the materials as the electron beam irradiation dose increases. Additionally, the presence in the IR spectra of irradiated composites of the absorption bands at 2920, 2850, 1456, 377, 810 and 720 cm^−1^ proves that the polymer chain in EPDM was not practically affected by electron irradiation even at higher doses (150 kGy). The results are in good agreement with previous literature studies which specify that when the scissioning polymer was blended with a crosslinked polymer, the resulting composites can retain its physical-mechanical characteristics at high absorbed doses [45]. In our case, EPDM is the polymer with a marked tendency to crosslink by irradiation and halobutyl rubber can degrade under electron irradiation [25,46,47].

**Figure 11:**
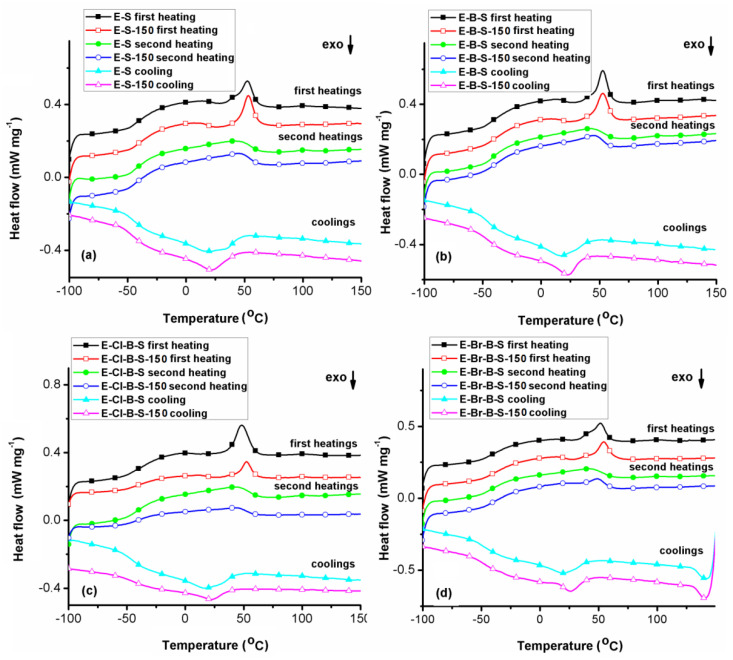
Differential scanning calorimetry (DSC) curves of initial and irradiated at 150 kGy samples: (**a**) E-S, (**b**) E-B-S, (**c**) E-Cl-B-S, (**d**) E-Br-B-S.

The DSC experiments were used to evaluate the changes between transition temperatures of non–irradiated and electron beam irradiated samples. The DSC traces of the initial samples and the electron beam irradiated at 150 kGy are shown in Figure 11. It is clear that these composites exhibited no noteworthy differences in transitions at higher irradiation doses. Specific calorimetric data extracted from the DSC curves are summarized in Table 3. From DSC scans it can observe that the glass transition temperature (*T*_g_) domain of non–irradiated structures ranges between −44 °C and −31 °C. These structures also exhibit a melting profile (*T*_m_) with a maximum located in the range 36–53 °C, depending on sample composition. The EPDM rubber composites exhibited a single glass transition temperature, even after electron beam irradiation, demonstrating an effective interfacial reaction between the components of the polymer matrix. The single glass transition temperature is an indicator of the good component miscibility [28,48]. The crystallization temperature (*T*_cr_) values of non-irradiated samples are located between 15 and 22 °C (Table 3). As can be seen from the DSC data, the *T*_g_ values corresponding to the irradiated samples increase, as a consequence of lower chain segments mobility, due to the crosslinking occurrence [49,50]. Additionally, the *T*_m_ and *T*_cr_ values shifted towards higher temperatures, accompanied by a decrease in melting (Δ*H*_m_) and crystallization (Δ*H*_cr_) enthalpies after electron beam irradiation. This fact indicates the presence of the crosslinking process and crystalline phase decreases during irradiation. Furthermore, the cold crystallization transitions (*T*_cc_), present on all first heating runs, are also shifted towards a higher temperature range and higher values for crystallization enthalpy (Δ*H*_cc_) were obtained after irradiation, suggesting an increase in the amorphous fraction as a result of longer crosslinked macromolecules. This point is in agreement with the FTIR data relating to the disappearance of the absorption band around 767 cm^−1^ due to the crystalline ethylene units during irradiation.

The TGA thermograms and their derivate curves (DTG) of the 150 kGy irradiated EPDM composites and the results are presented in Table 4 for all EPDM/butyl rubber/nanosilica composites. The thermograms and the first derivative curves of the initial samples and those irradiated at 150 kGy exhibited no visually discernible differences. However, some differences were observed only from the extracted thermal parameters, such as temperatures corresponding to 5 wt.%, (*T*_5%_), 10 wt.% (*T*_10%_), 30 wt.% (*T*_30%_) and 50 wt.% (*T*_50%_) mass loss, maximum peak of decomposition (*T*_max_); individual mass loss for each thermal degradation stage (*W*) and residual char mass remained after thermal degradation at 700 °C (*R*).

It can be seen that all composites showed one thermal main decomposition stage, corresponding to the decomposition of EPDM and EPDM/butyl rubber mixture in the composites (Figure 11). A single thermal degradation stage confirms the good miscibility between components [51,52]. The thermal degradation was shifted to higher temperatures (416–445 °C) (*T*_5%_) for the samples irradiated at a dose of 150 kGy as compared to the non-irradiated composites (412–438 °C). The higher thermal decomposition temperature range of the irradiated composites is also an indication of crosslinking phenomena occurrence during irradiation in agreement with DSC data. Additionally, for the irradiated composites, the *T*_10%_, *T*_30%,_ *T*_50%_ and *R* values increased and *W* values decreased, further indicating the occurrence of crosslinking phenomena.

**Figure 12:**
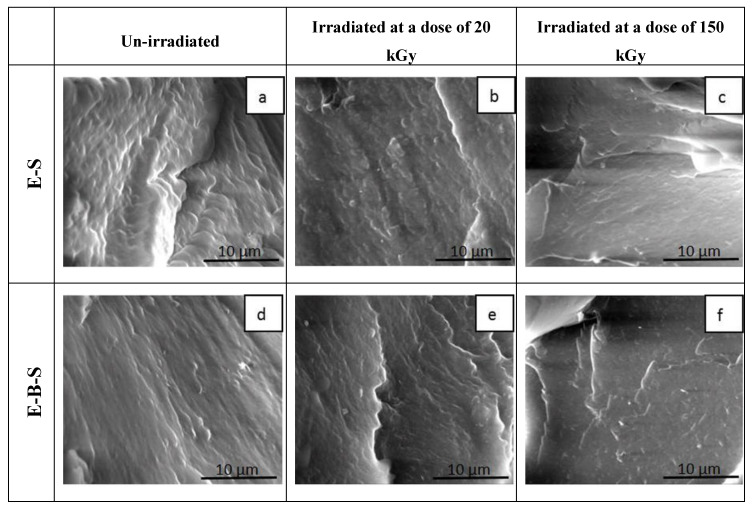
Micrographs of fractured surfaces of EPDM/rubber/nanosilica composites before and after irradiation (10,000 X magnification): (**a**) E-S 0 kGy, (**b**) E-S 20 kGy, (**c**) E-S 150 kGy, (**d**) E-B-S 0 kGy, (**e**) E-B-S 20 kGy, (**f**) E-B-S 150 kGy.

The morphology of the fractured surface of the EPDM rubbers/nanosilica composites was studied by means of SEM. The SEM images of the fractured surfaces of irradiated samples at 20 kGy and 150 kGy, respectively, and non-irradiated E-S and E-B-S composites are shown in Figure 12. The micrographs of the non-irradiated EPDM composites as shown in Figure 12a,d reveal a very smooth surface with some embedded small particles of nanosilica filler. In the case of E-B and E-B-S samples, it can be noticed that the composites have a homogeneous structure which means that the nanosilica particles are uniformly distributed in the polymer matrix. The smooth appearance of fractured surfaces indicates that there are no filler agglomerations that could decrease the mechanical strength of the composites. Figure 12b,e show micrographs of E-S and E-B-S after irradiation at 20 kGy. A comparison between these micrographs shows that the surface becomes more uniform and smooth after irradiation and this may be attributed to crosslinking by irradiation. Increasing the irradiation dose at 150 kGy the polymer matrix surface becomes smoother (Figure 12c,f). This behavior can be determined by the decrease in interfacial tension between components leading to a stable morphology by interfacial adhesion and these materials present superior mechanical properties as compared to EPDM/butyl rubber composites.

**Figure 13:**
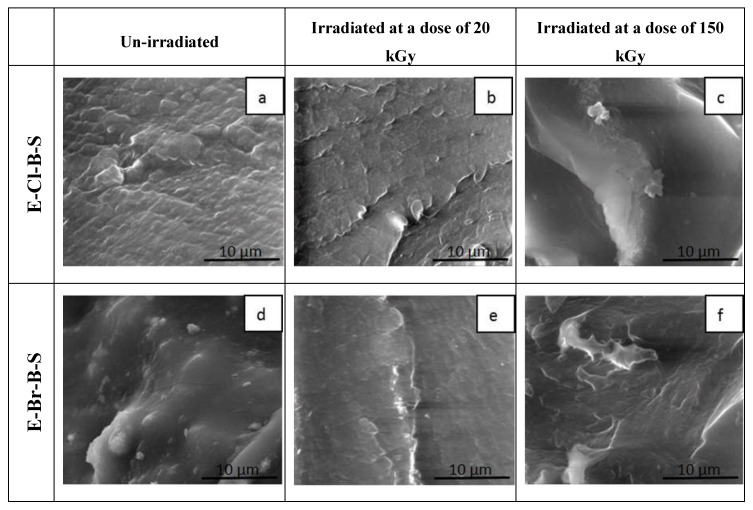
SEM images of fractured surfaces of EPDM/rubber/nanosilica composites before and after irradiation (10,000 X magnification): (**a**) E-Cl-B-S 0 kGy, (**b**) E-Cl-B-S 20 kGy, (**c**) E-Cl-B-S 150 kGy, (**d**) E-Br-B-S 0 kGy, (**e**) E-Br-B-S 20 kGy, (**f**) E-Br-B-S 150 kGy).

The E-Cl-B-S and E-Br-B-S samples have a different morphology and distinct microdomains formed by small agglomerations of nanosilica particles can be observed (Figure 13). After irradiation with a dose of 20 kGy, a decrease in the roughness of the E-Cl-B-S and E-Br-B-S composites can be displayed (Figure 13b,e). The difference between the morphology of irradiated EPDMS composites consists of the shape of ridgelines. The ridgelines indicate the direction of cracks propagation and are correlated with the compatibility between nanosilica-filler and EPDM rubbers. The micrographs of irradiated samples with a dose of 150 kGy (Figure 13c,f) show much more prominent ridgelines due to the fact that part of the filler is not well dispersed and forms agglomerations of nanosilica particles. Additionally, the reduction of the surface roughness at a higher irradiation dose (150 kGy) can indicate an increase in miscibility between phases by crosslinking [53].

The surface behavior of the samples was investigated through measurements of contact angles against three test solvents (water, formamide, and diiodomethane) and the results are given in Table 5. The addition of the inorganic phase into composites changes the properties of the material but also can influence the surface properties such as surface wettability. The control sample, which does not contain butyl rubber and nanosilica, exhibits a contact angle of 92.3° [19].

Adding 5 phr of nanosilica to rubber composite, an increase in the contact angle was observed for the three test solvents due to the engagement of silanol groups from nanosilica surface in the formation of some physical interactions with carbonyl groups from TMPT or CH_3_ groups from rubbers [54]. For example, for sample E-B-S an increase of 14° was found in water. The contact angle values of EPDM composites have an ascending trend relating to the increase in the irradiation dose (Table 5), the hydrophobic character maintaining after electron beam irradiation. The contact angle calculated between the polar liquid drops (water and formamide) and the surface of the E-B-S sample registered the highest values both before and after irradiation treatments probably due to the richness of hydrophobic methyl groups. Compared to the E-B-S, the chlorinated and brominated butyl rubber-based samples showed lower values of θ due to the absence of one CH_3_ group which is replaced with one Cl or one Br molecule in the butyl rubber chemical structure.

As a general observation for all investigated compositions in this study, the total surface free energy (γlv^TOT^) of non-irradiated samples is significantly decreased after electron beam irradiation due to the decrease in both polar and dispersion components of the surface energy (Table 6). This trend has also been reported in our previous study [19], when the sample did not contain nanosilica. However, some changes occurred in the polarities of the samples embedded with hydrophilic silica. Thus, the addition of nanosilica filler in the non-irradiated EPDM rubber control sample leads to a decrease in the polarity of the sample surface from 24.63% to 15.86% (Table 6). After electron beam irradiation at a dose of 20 kGy, the presence of filler into the EPDM rubber induces a slight increase in the polarity from 11.15% to 12.17% and a more substantial increase from 4.76% to 18.57% after the irradiation with 150 kGy. These results demonstrate that electron beam irradiation changes the surface properties of silica, being more compatible with EPDM chains and more uniformly dispersed in the rubber matrix by favoring the polymer/filler physical interaction and reducing the interparticle forces.

**Figure 14:**
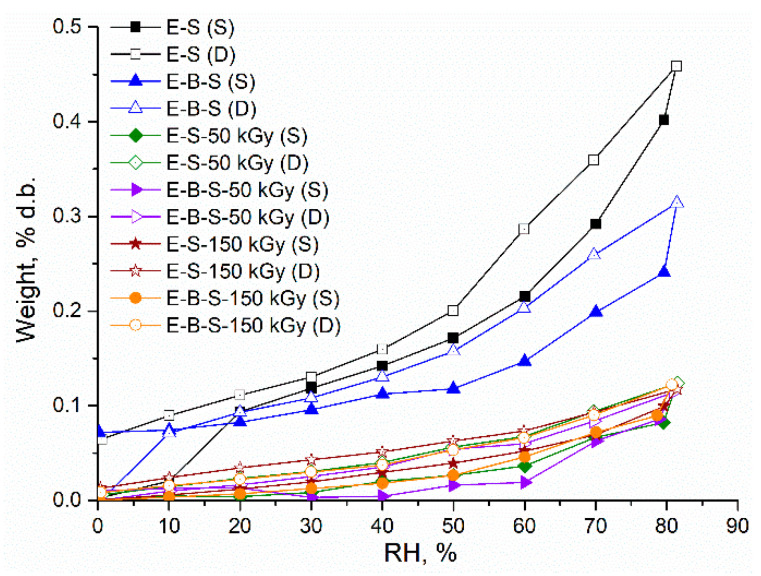
Dynamic vapor sorption isotherms for tested composites E-S; E-B-S.

**Figure 15:**
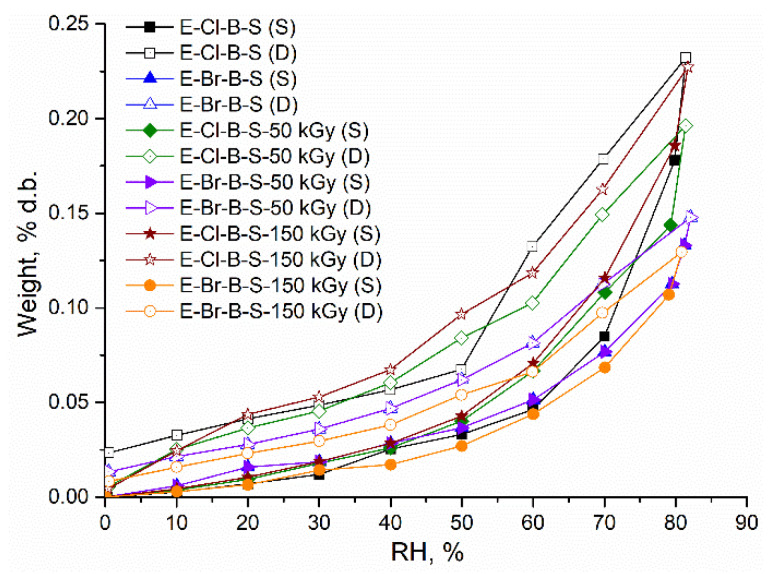
Dynamic vapor sorption isotherms for tested composites E-Cl-B-S; E-Br-B-S.

The sorption/desorption isotherm loops are presented in Figure 14 and Figure 15. According to IUPAC classification, sorption–desorption loops can be associated with type III isotherms, where adsorbate–sorbent interactions are smaller as compared to the adsorbate–adsorbate interactions. These isotherms can be found for dispersing, nonporous or macroporous solids. The small hysteresis loops appear due to slower desorption rates in comparison with the sorption rate.

Average pore size can be estimated according to Equation (7), where *r_pm_* is the average pore size, *A* represents BET surface area, *n* is the percentage uptake, *ρ_a_* denotes the solvent phase density (Table 7).

(7) $$r_{pm}=\frac{2n}{100\cdot \rho_{a}\cdot A}$$

For the non-irradiated samples, the following order of sorption capacity was established: E-S > E-B-S > E-Cl-B-S > E-Br-B-S. This decrease in the sorption capacity is accompanied by a noticeable increase in the average pore size due to the presence of the polar entities in composites. It is noticed that the electron beam irradiated samples showed a lower water uptake tendency as compared to the corresponding non-irradiated composites. In the case of E-S and E-B-S series (Figure 14), the irradiation dose greatly affects the uptake behavior by reducing the sorption capacity and drastically decreasing the BET area (specific surface area). Contrary, the polar entities gave radiation stability, thus small variations of the sorption capacity of the BET area can be seen for E-Cl-B-S and E-Br-B-S samples (Figure 15). According to the SEM data (Figure 12), the crosslinking by irradiation determines a smoother surface of the composite generating thus a matrix with relatively lower free volume sites for water transport. Additionally, good interfacial bonding between rubber and nanosilica leads to slower water absorption.

## 4. Conclusions

In conclusion, the electron beam irradiation process of the prepared composites was beneficial to the mechanical properties of these materials. However, at higher irradiation doses, the elongation at break and elongation set decreased with an increase in the irradiation dose, whereas the tensile strength and elasticity presented an increase up to a dose of 50 kGy, and then their values decreased. As noted in the results, the values of p_0_/q_0_ ratio (Charlesby–Pinner equation) are smaller than 1, suggesting that the crosslinking of polymer chains is the predominant process under electron beam irradiation. The DSC thermograms present a single glass transition temperature due to the miscibility of components in composites. The thermal decomposition of the electron beam irradiated EPDM composites occurred to higher temperatures due to the crosslinking of the polymer chains during irradiation. The contact angle determinations indicated the hydrophobic character of the composite surface and the hydrophobicity increases as the irradiation dose increases. The water uptake presented decreasing accessibility of the solvent in the electron beam irradiated samples.

## Figures and Tables

**Table 1:** Formulation of the Ethylene–propylene–diene terpolymer (EPDM)/butyl(halobutyl) rubber/nanosilica composites.

Ingredients (phr *)	Sample Code			
	E-S	E-B-S	E-Cl-B-S	E-Br-B-S
EPDM (E)	100	95	95	95
TMPT	3	3	3	3
Irganox	0.5	0.5	0.5	0.5
Nanosilica	2	2	2	2
Butyl rubber (B)	-	5	-	-
Cl-butyl rubber (Cl-B)	-	-	5	-
Br-butyl rubber (Br-B)	-	-	-	5
Total	105.5	105.5	105.5	105.5

* Parts per hundred rubber.

**Table 2:** V_rf_ and V_ro_ /V_rf_ values of EPDM/butyl rubber/nanosilica composites in toluene.

Sample	Absorbed Dose (kGy)	V_rf_	V_ro_/V_rf_
E-S	20	0.1601	1.0295
	50	0.2646	0.6228
	100	0.3518	0.4684
	150	0.3856	0.4273
E-B-S	20	0.1609	1.0208
	50	0.2571	0.6389
	100	0.3415	0.4810
	150	0.3708	0.4429
E-Cl-B-S	20	0.1470	1.1886
	50	0.2000	0.8738
	100	0.3013	0.5800
	150	0.3448	0.5068
E-Br-B-S	20	0.1875	1.0590
	50	0.2307	0.8607
	100	0.3261	0.6088
	150	0.3613	0.5496

**Table 3:** Data extracted from the DSC curves of the EPDM rubber composites.

Sample.	*T*_g1_ (°C)	*T*_m1_ (°C)	Δ*H*_m1_ (J g^−1^)	*T*_g2_ (°C)	*T*_m2_ (°C)	Δ*H*_m2_ (J g^−1^)	*T*_cr_ (°C)	Δ*H*_cr_ (J g^−1^)	*T*_cc_ (h1) (°C)	Δ*H*_cc_ (J g^−1^)
E-S	−39	52	8.71	−44	40	17.26	15	−23.84	27	−1.47
E-S-150	−37	54	8.27	−42	45	16.18	22	−21.22	31	−2.37
E-B-S	−40	53	9.07	−41	36	13.69	16	−13.41	25	−2.51
E-B-S-150	−37	53	9.44	−39	45	13.16	23	−13.73	25	−2.84
E-Cl-B-S	−36	48	13.79	−43	42	12.92	17	−16.82	–	–
E-Cl-B-S-150	−31	53	5.17	−40	47	7.25	22	−8.96	28	−1.16
E-Br-B-S	−40	52	7.42	−39	41	11.88	20	−13.5	27	−1.40
E-Br-B-S-150	−34	55	6.80	−36	49	11.17	26	−12.74	29	−1.86

h_1_—first heating scan; h_2_—second heating scan; *T*_g1_—glass transition domain temperature recorded on the first heating scan; *T*_g2_—glass transition domain temperature recorded on the second heating scan; *T*_m1_—melting temperature recorded on the first heating scan; *T*_m2_—melting temperature recorded on the second heating scan; *T*_cr_—crystallization temperature; *T*_cc_—cold crystallization temperature; Δ*H*_m1_—enthalpy of the melting profile recorded on the first heating scan; Δ*H*_m2_—enthalpy of the melting profile recorded on the second heating scan; Δ*H*_cr_—crystallization enthalpy; Δ*H*_cc_—cold crystallization enthalpy.

**Table 4:** Summary of thermogravimetric analyses (TGA) results for the EPDM composites.

Sample	*T*_5%_ (°C)	*T*_max_ (°C)	*W* (%)	*T*_10%_ (°C)	*T*_30%_ (°C)	*T*_50%_ (°C)	*R* (%)
E-S	438	473	96	448	461	470	2.85
E-S-150	445	475	94	452	465	472	4.07
E-B-S	417	473	92	430	456	465	3.44
E-B-S-150	420	474	94	432	458	468	3.88
E-Cl-B-S	413	470	96	431	456	468	3.31
E-Cl-B-S-150	419	472	94	433	457	475	4.75
E-Br-B-S	412	471	94	431	455	466	4.36
E-Br-B-S-150	416	472	93	433	457	467	4.96

*T*_max_—maximum decomposition temperature rate; *T*_5%_, *T*_10%_, *T*_30%_, *T*_50%_—temperatures corresponding to 5 wt.%, 10 wt.%, 30 wt.% and 50 wt.% mass losses; W—mass loss per each stage; R—residual char mass at 700 ^o^C.

**Table 5:** Contact angles (θ) with test liquids of sample surfaces before and after electron beam irradiation.

Sample		Contact Angle (Ɵ) Values		
		Water	Formamide	Diiodomethane
0 kGy	E-S	102.4	90.4	51.6
	E-B-S	106.2	94.9	58.2
	E-Cl-B-S	101.1	92.3	73.5
	E-Br-B-S	98.9	92.2	61.3
20 kGy	E-S	109.6	97.1	67.7
	E-B-S	111.6	104.2	77.7
	E-Cl-B-S	109.3	97.8	78.4
	E-Br-B-S	108.8	100.6	79.0
150 kGy	E-S	112.2	104.7	80.0
	E-B-S	113.3	108.8	86.8
	E-Cl-B-S	111.9	106.6	88.6
	E-Br-B-S	110.4	105.8	87.2

**Table 6:** Surface free energy and polar and dispersive components values of samples before and after electron beam irradiation; all values are expressed in mN/m, excepting P.

Sample		γ_sv_^LW^	γ_sv_^+^	γ_sv_^-^	γ_sv_^AB^	γ_lv_^TOT^	P%
0 kGy	E-S	33.38	2.6328	3.76	6.29	39.67	15.86
	E-B-S	29.61	2.5962	3.15	5.72	35.33	16.18
	E-Cl-B-S	20.94	0.4338	5.04	2.96	23.90	12.37
	E-Br-B-S	27.83	2.0860	7.05	7.67	35.50	21.60
20 kGy	E-S	24.15	1.3893	2.02	3.35	27.50	12.17
	E-B-S	18.69	1.5495	3.25	4.49	23.18	19.36
	E-Cl-B-S	18.35	0.3904	2.21	1.86	20.21	9.19
	E-Br-B-S	18.01	0.8119	3.48	3.36	21.37	15.73
150 kGy	E-S	17.50	1.2929	3.08	3.99	21.49	18.57
	E-B-S	14.15	1.1605	3.83	4.22	18.37	22.95
	E-Cl-B-S	13.33	0.6330	3.75	3.08	16.41	18.76
	E-Br-B-S	14.00	0.7549	4.43	3.66	17.66	20.71

P% = Polarity calculated as γ_sv_^AB^/ γlv^TOT^ * 100 (percentage of polar component in overall surface free energy).

**Table 7:** Main data evaluated from sorption isotherms of tested samples.

Sample	Sorption Capacity (% d.b.)	*r_pm_*, (nm)	BET Data	
			Area (m^2^ g^−1^)	Monolayer (g g^−1^)
E-S-0 *	0.400	0.64	12.6	0.00350
E-S-50	0.080	2.43	0.66	0.00019
E-S-150	0.100	1.73	1.16	0.00033
E-B-S-0	0.240	0.83	5.80	0.00160
E-B-S-50	0.114	4.57	0.50	0.000001
E-B-S-150	0.122	2.75	0.89	0.000254
E-Cl-B-S-0	0.177	4.80	0.74	0.0021
E-Cl-B-S-50	0.143	1.70	1.69	0.000482
E-Cl-B-S-150	0.185	1.98	1.87	0.000535
E-Br-B-S-0	0.120	2.64	0.912	0.00026
E-Br-B-S-50	0.126	2.79	0.905	0.000301
E-Br-B-S-150	0.107	2.41	0.890	0.000256

* 0, 50 and 150 stands for absorbed radiation dose (kGy).
